# Transcriptome profiling of tobacco (*Nicotiana tabacum*) pollen and pollen tubes

**DOI:** 10.1186/s12864-017-3972-3

**Published:** 2017-08-07

**Authors:** Lei Liu Conze, Sofia Berlin, Aude Le Bail, Benedikt Kost

**Affiliations:** 1 Department of Plant Biology, Swedish University of Agricultural Sciences, Uppsala BioCenter, Linnean Centre for Plant Biology, Uppsala, Sweden; 2 Cell Biology Division, Department of Biology, Friedrich Alexander University, Erlangen/Nuremberg, Germany

**Keywords:** Tobacco, Pollen, Pollen tube growth, Polar cell expansion, RNA-seq

## Abstract

**Background:**

Pollen tube growth is essential for plant reproduction and represents a widely employed model to investigate polarized cell expansion, a process important for plant morphogenesis and development. Cellular and regulatory mechanisms underlying pollen tube elongation are under intense investigation, which stands to greatly benefit from a comprehensive understanding of global gene expression profiles in pollen and pollen tubes. Here, RNA sequencing technology was applied to de novo assemble a *Nicotiana tabacum* male gametophytic transcriptome and to compare transcriptome profiles at two different stages of gametophyte development: mature pollen grains (MPG) and pollen tubes grown for six hours in vitro (PT6).

**Results:**

De novo assembly of data obtained by 454 sequencing of a normalized cDNA library representing tobacco pollen and pollen tube mRNA (pooled mRNA isolated from mature pollen grains [MPG] and from pollen tubes grown in vitro for 3 [PT3] or 6 [PT6] hours) resulted in the identification of 78,364 unigenes. Among these unigenes, which mapped to 24,933 entries in the Sol Genomics Network (SGN) *N. tabacum* unigene database, 24,672 were predicted to represent full length cDNAs. In addition, quantitative analyses of data obtained by Illumina sequencing of two separate non-normalized MPG and PT6 cDNA libraries showed that 8979 unigenes were differentially expressed (differentially expressed unigenes: DEGs) between these two developmental stages at a FDR q-value of <0.0001. Interestingly, whereas most of these DEGs were downregulated in PT6, the minor fraction of DEGs upregulated in PT6 was enriched for GO (gene ontology) functions in pollen tube growth or fertilization.

**Conclusions:**

A major output of our study is the development of two different high-quality databases representing the tobacco male gametophytic transcriptome and containing encompassing information about global changes in gene expression after pollen germination. Quantitative analyses of these databases 1) indicated that roughly 30% of all tobacco genes are expressed in the male gametophyte, and 2) support previous observations suggesting a global reduction of transcription after pollen germination. Interestingly, a small number of genes, many of which predicted to function in pollen tube growth or fertilization, were found to be upregulated in elongating pollen tubes despite globally reduced transcription.

**Electronic supplementary material:**

The online version of this article (doi:10.1186/s12864-017-3972-3) contains supplementary material, which is available to authorized users.

## Background

Mature pollen and growing pollen tubes represent two different developmental stages of the haploid male gametophyte of flowering plants, which produces two sperm cells. During fertilization, one sperm cell fuses with an egg cell to generate a zygote, whereas the other fuses with a central cell to form a primary endosperm cell, a phenomenon known as double fertilization. To enable fertilization, the two sperm cells are transported to an ovary by a pollen tube, which grows in a polarized fashion through female flower tissues after it is formed by a germinating pollen grain on the surface of the stigma. Pollen germination and pollen tube growth are thus essential for the sexual reproduction of flowering plants.

By a process known as tip growth, pollen tubes elongate extremely rapidly at rates of several μm per minute [1, 2]. This process is widely employed as an experimental model to investigate cellular and molecular mechanisms underlying polar cell expansion, which is essential for morphogenesis during plant development [3]. Pollen tube tip growth depends on massive apical secretion of cell wall material, which requires the actin cytoskeleton and needs to be balanced by endocytic membrane recycling [1, 2, 4]. The different cellular structures and processes that mediate tip growth are regulated and coordinated by a complex signaling network, in which Rac/ROP (Rho of plant) family small GTPases [5, 6], Ca^2+^ [7–9] and proton [10] fluxes across the plasma membrane, as well as reactive oxygen species (ROS) [11] appear to play key roles. Rapid pollen germination and subsequent pollen tube growth at high rates appear to be enabled by a massive accumulation of mRNA, proteins and ribosomes in pollen during maturation [12–17]. Although cell and molecular biological as well as biochemical research has identified a large number of proteins with important functions in pollen germination and/or pollen tube growth, in many cases the precise molecular identity and/or functions of these proteins remain to be clarified. To address these open questions and to generally enhance our understanding of the genetic and molecular control of different male gametophyte functions (pollen germination, pollen tube growth, fertilization), it is essential to establish high-quality databases representing the transcriptome of the male gametophyte and providing information about global changes in gene expression during its development.

One of the most commonly used model systems to study pollen tube tip growth is tobacco (*Nicotiana tabacum*), because pollen tubes of this species grow exceptionally well in vitro and can easily be cultured in quantities sufficient for biochemical analysis as well as RNA isolation. Tobacco is an allotetraploid (2n = 4× = 48) plant with a large (approximately 4.5 Gb) and complex genome that contains a significant proportion (> 70%) of repeats [18–20]. First drafts of the tobacco genome sequence have recently become available [20]. Although a high-quality tobacco male gametophyte transcriptome database has been missing to date, valuable information concerning global gene expression at different stages of tobacco male gametophyte development has been obtained based on microarray analyses using the Agilent 44 K tobacco gene chip. These analyses indicated that 13,966 genes are expressed in mature pollen [21], 14,100 genes in pollen tubes grown for 4 h [21] and 14,420 genes in pollen tubes grown for 24 h [22]. As the number of genes in the tobacco draft genomes was recently estimated to be somewhere between 81,000 and 94,000 [20], the 44 K gene chip can obviously only represent roughly 50% of all tobacco genes. With the aim to get a more complete picture of the genetic basis of tobacco pollen germination and pollen tube growth, we decided to employ RNA sequencing (RNA-seq) technology. As this technology is not dependent on any prior information and allows effective de novo transcriptome assembly in the absence of a reference genome sequence, it is particularly suitable for characterizing global gene expression in tobacco and other organisms with large and complex genomes, for which no or only draft sequences are available. Furthermore, RNA-seq is considered the best currently available technique to detect not only the expression of novel genes, but also of genes expressed a very low or extremely high levels [23].

In the study presented here, we first developed a high-quality tobacco male gametophytic transcriptome sequence database based on de novo assembly of data obtained by 454 sequencing of a normalized cDNA library prepared from pooled RNA, which was isolated from the male gametophyte at three developmental stages: mature pollen grains (MPG) as well as in vitro germinated pollen tubes grown for 3 (PT3) and 6 h (PT6). 454 sequencing generates relatively long reads (ca. 650 bp in average) and was therefore the method of choice for this purpose. MPG, PT3 and PT6 RNA was pooled with the aim to develop a single sequence database that represents the male gametophyte transcriptome (genes expressed in mature pollen and/or in growing pollen tubes) as completely as possible. Library normalization was performed to increase the chance of weakly expressed genes to be represented in the transcriptome database. Analysis of different indicators confirmed that the transcriptome database generated is of high quality and provides an encompassing coverage of the tobacco male gametophyte transcriptome. A comparison of our transcriptome database with the draft tobacco genome indicated that consistent with previous estimations based on microarray data [21, 22] roughly 30% of all tobacco genes are expressed in the male gametophyte.

After pooling MPG, PT3 and PT6 RNA, as well as library normalization, the transcriptome database generated based on 454 sequencing of course does not contain any information concerning levels of gene expression at different developmental stages. Two additional non-normalized 3′ fragment cDNA libraries were therefore separately prepared from male gametophytic RNA isolated either at the MPG or the PT6 stage. These libraries were subjected to Illumina sequencing, which generates large numbers of relatively short reads (ca. 100 bp) and is therefore optimally suited for the quantification of gene expression. This approach enabled the development of a MPG/PT6 expression database, which contains encompassing information concerning the expression level at the MPG and PT6 stage of genes represented in the transcriptome sequence database assembled based on 454 sequencing. Using the MPG/PT6 expression database, extensive analyses of global changes in gene expression after tobacco pollen germination were performed, which confirmed previous observations that transcriptional activity is generally downregulated during this process. Interestingly, these analyses also resulted in the identification of a group of genes, which against this trend are upregulated in elongating pollen tubes and in many cases are predicted to function in cellular processes important for tip growth. The MPG/PT6 expression database can of course also be employed to investigate the regulation of selected individual, or groups of, genes during pollen germination. Transcript levels of 8 selected genes that are strongly up- or downregulated after pollen germination according to the MPG/PT6 expression database, and that have predicted functions in processes important for tip growth, were also subjected to quantitative real-time PCR (qRT-PCR) analysis, which confirmed the high quality of the MPG/PT6 expression database.

## Results

### 454 sequencing and de novo assembly

**Fig. 1:**
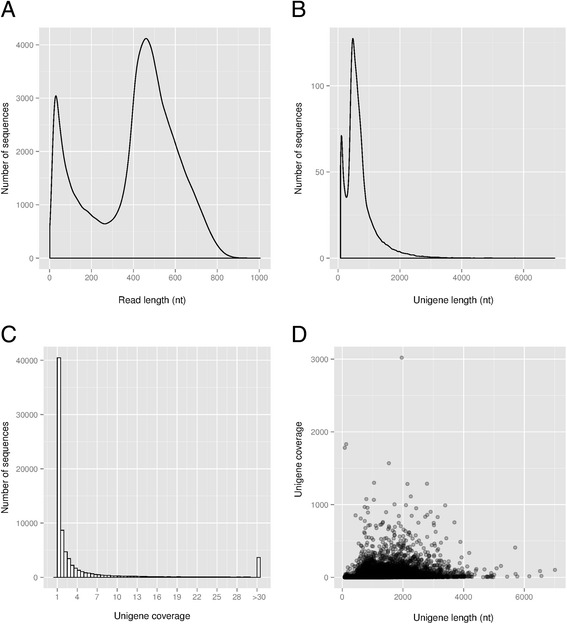
Tobacco male gametophyte transcriptome sequencing and assembly: overview. **a** Density plot of the 454 sequencing read length after filtering and trimming adapters. **b** Density plot of the length of de novo assembled unigenes. **c** Histogram of the sequencing coverage of the assembled unigenes. Coverage values greater than 30× have been binned. **d** Density scatterplot showing the relationship between unigene length and coverage. Points with a high local density are darker

To sequence the tobacco male gametophytic transcriptome, equal amounts of mRNA isolated at the MPG, PT3 and PT6 stages of development were pooled. A normalized cDNA library was synthesized from the pooled mRNA by Eurofins MWG GmbH and subjected to a full plate run on the 454 GS FLX platform. The 454 sequencing generated a total of 1,419,427 reads, with an average sequence length of 643 nt (Table 1). After stringent quality and adapter trimming, 1,417,567 clean reads remained with an average length of 406 nt (Table 1, Fig. 1a, NCBI Sequence Read Archive [SRA] accession number SRP093461). These clean sequencing reads were assembled using the MIRA assembler v3.4 [24]. To reduce assembly errors and to increase contig size, contigs and singletons were clustered in four separate cycles in MIRA and one additional cycle in CAP3 [25]. In the resulting assembly, 90% of the clean reads were either present as 139,059 singletons or incorporated into 42,316 contigs with an average sequence coverage of 13.35× (Table 2). By setting a length cut-off of 80 nt, our final assembly consisted of 76,364 sequences (i.e. retained singletons plus contigs, Table 2) hereafter referred to as unigenes (Additional file 1). These unigenes have an average length of 683 nt and the average sequence coverage was 7.83× (Table 2, Fig. 1b). The distribution of unigene coverage was highly left-skewed towards low coverage with an extremely long tail (maximum coverage is 3,019×; Fig. 1c and d), which is typical for a normalized library and suggests that cDNA normalization was effective [26].

**Table 1:** Sequence statistics

	Raw reads	Cleaned reads
Number of sequences	1,419,427	1,417,567
Mean length (nt)	643	406
Median size (nt)	605	451
N50 size (nt)	637	512
Range in length (nt)	55–1601	1–1004

**Table 2:** De novo assembly summary statistics

	MIRA + CAP3
Number of reads assembled into contigs	1,278,508
Number of contigs	42,316
Average length of contigs (nt)	886
Range of contig lengths (nt)	40–7008
Average coverage of contigs (reads/base)	13.35
Number of singletons	139,059
Average length of singletons (nt)	127
Range of singleton lengths (nt)	1–1004
Number of large contigs (> 80 nt)	41,427
Number of large singletons (>80 nt)	34,937
Total number of unigenes	76,364
Average coverage of unigenes (reads/base)	7.83
Average length of unigenes (nt)	683

### Number of expressed genes in mature pollen and pollen tubes

To estimate the number of genes represented in the tobacco male gametophytic transcriptome generated as described in the previous paragraph, the de novo assembled 76,364 unigenes were queried by BLAST against the *N. tabacum* unigene databases from SGN [27] and were found to match 24,933 of all unique entries in these databases. Provided that all 84,602 entries in the SGN unigene databases represent single genes and that this database effectively covers the tobacco genome, an assumption that is consistent with the 81,000 and 94,000 genes identified in tobacco draft genome sequences [20], about 30% of all *N. tabacum* genes therefore appear to be expressed in pollen and pollen tubes (Table 3, Additional file 2: Table S1).

**Table 3:** BLAT results of 454 assembly against the SGN tobacco unigene database

Database name	Database size	Nr. of matched database entries	% of matched database
SGN tobacco unigenes	84,602	24,933	29.5%

### Reference assemblies

**Fig. 2:**
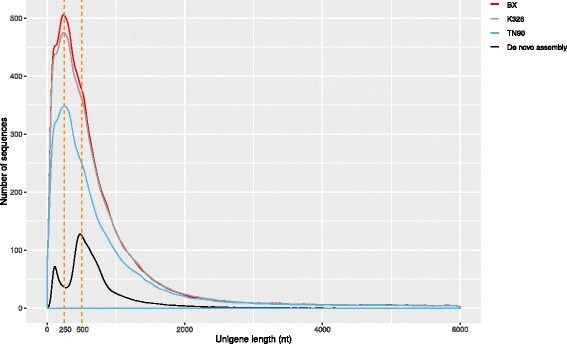
Comparison of contig number and size between de novo and reference assemblies. Density plot of unigene length

We compared our de novo assembly with reference assemblies against the three available draft tobacco genomes: *N. tabacum* TN90, *N. tabacum* K326 and *N. tabacum* Basma Xanthi [20]. Of the 1,417,567 clean 454 sequencing reads, ~95% displayed a significant match to each of the three draft genomes. The reference-based assemblies were performed by Cufflinks [28] and for each draft genome we obtained more than 300,000 contigs. However, these contigs mapped only to roughly the same number (ca. 25,000) of unique entries in the SGN *N. tabacum* unigene database as the 76,364 contigs obtained by de novo assembly (see previous paragraph). Furthermore, not only the number but also the size of the contigs generated by each of the reference assemblies was unrealistically large (Fig. 2). About 1% of these contigs were longer that 5000 nt and therefore appear likely to be incorrectly assembled. By contrast, after de novo assembly only 0.01% of all contigs exceeded this size limit. Together, these observations strongly suggest that the reference assemblies suffer from substantially higher mis-assembly rates and higher redundancy as compared to the de novo assembly. The de novo assembly was therefore employed for all further analyses discussed below.

### Completeness of the de novo assembled transcriptome

To get an estimate of how completely the assembled unigenes cover the tobacco male gametophyte transcriptome, they were queried against two different groups of exceptionally highly conserved genes [29]: 1) the “ultra-conserved orthologous sequences” (UCOS) across eukaryotes and 2) the “shared single copy genes” of *Arabidopsis thaliana*, *Populus trichocarpa*, *Vitis vinifera*, and *Oryza sativa* (APVO), which was extracted from the PlantTribes database [30–32]. We found that the assembled unigenes mapped to 316 of 357 (89%) UCOS sequences and to 646 of 983 (66%) APVO sequences, which strongly suggests that these unigenes cover a major proportion of tobacco male gametophyte transcriptome.

### Full-length cDNA prediction

**Fig. 3:**
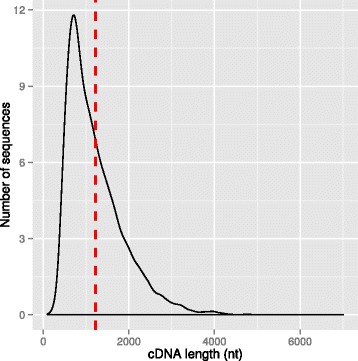
Density plot of the length of putative full-length cDNA sequences identified among de novo assembled unigenes

To identify full-length protein-coding cDNA sequences among the unigenes, we searched for sequences with complete open reading frames (ORFs) using the TargetIdentifer online tool [33]. Among the 76,364 unigenes, 24,672 full-length cDNA sequences ranging from 97 nt to 7008 nt with an average length of 1216 nt were identified (Fig. 3, Additional file 3: Table S2).

### Comparative analysis of gene expression in pollen and pollen tubes

**Fig. 4:**
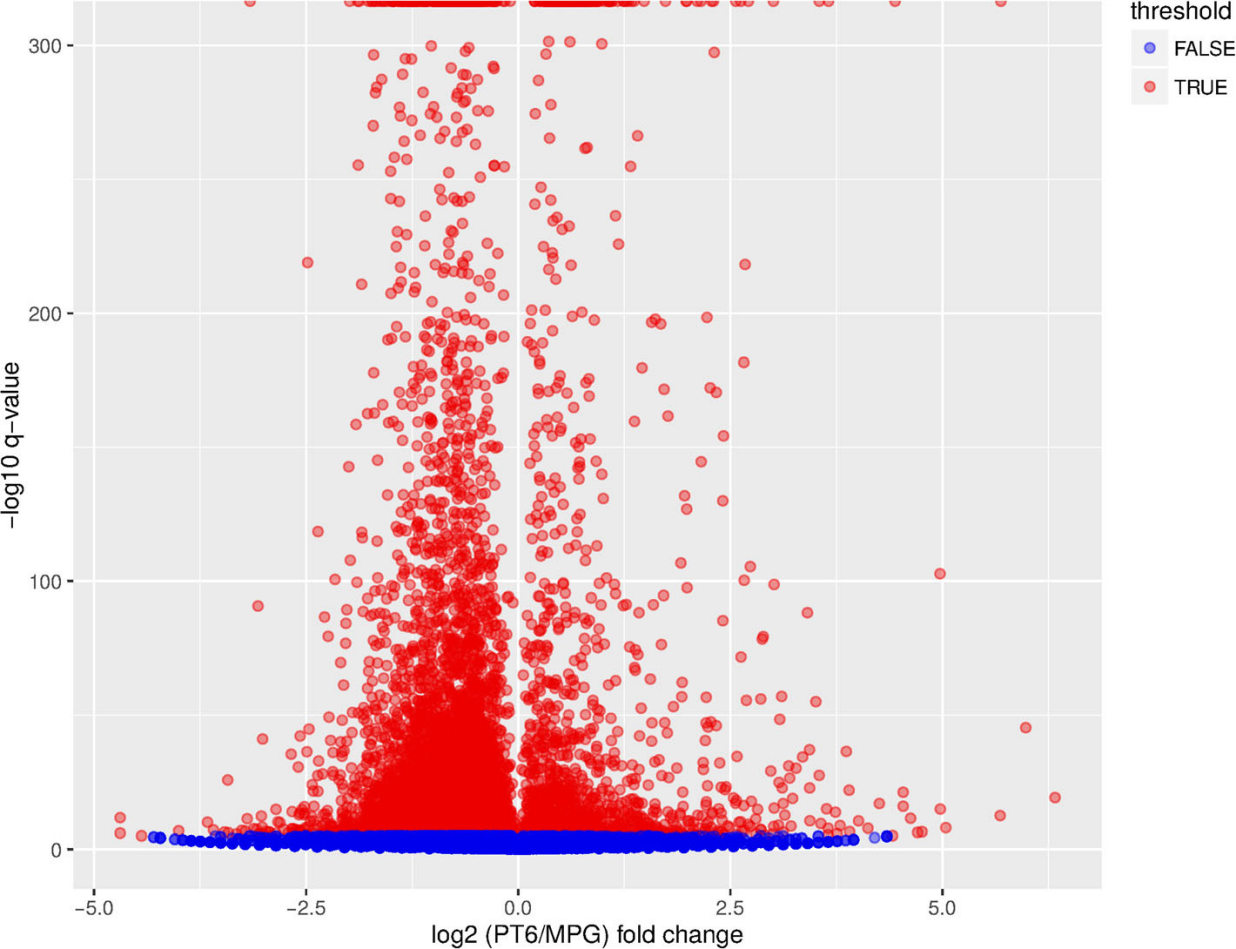
Volcano plot displaying unigenes differentially expressed between MPG and PT6. Red dots represent differentially expressed unigenes (false discovery rate [FDR] q < 0.0001). y-axis: -log10 *p*-value, x-axis: log2 fold change value

**Fig. 5:**
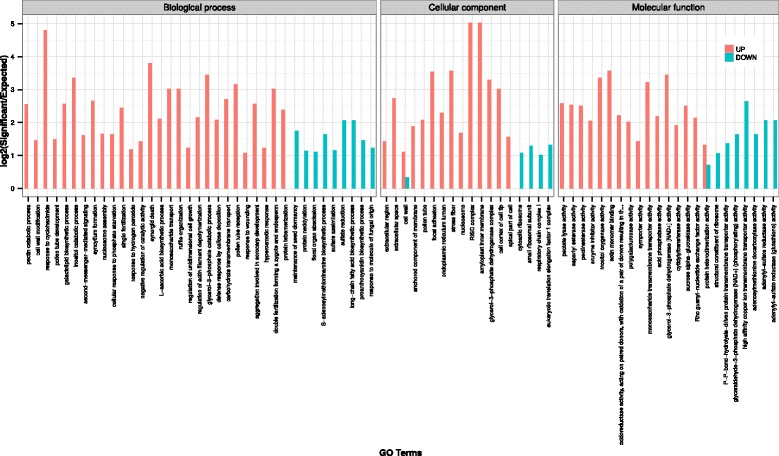
Top GO (Gene Ontology) categories enriched among unigenes differentially expressed between MPG and PT6 (DEGs). *Red* and *blue* bars represent GO categories enriched among upregulated or downregulated DEGs, respectively, as compared to all unigenes subjected to DEG analysis. x-axis: top GO categories (log2[Significant/Expected] >1; *P* < 0.01) of differentially expressed genes (q < 0.0001). y-axis: log2 of number of annotated genes (Significant) vs. expected number of annotated genes (Expected) in each GO category listed

In order to gain insights into the genetic control of pollen germination and pollen tube growth, the Illumina technology was used to sequence separate non-normalized 3′-fragment cDNA libraries prepared from MPG and PT6 mRNA samples. More than 50 million clean sequencing reads were generated from each library (NCBI Sequence Read Archive [SRA] accession number SRP093461) and roughly 98% of all obtained reads could be mapped by BLAST to the de novo assembled unigenes (Table 4). MPG and PT6 sequencing reads mapped to 44,012 and 43,810 unigenes, respectively. Unigenes differentially expressed between MPG and PT6 were identified based on sequence coverage using the R package DEGSeq [34]. A total of 8979 unigenes were found to be significantly differentially expressed (q-value <10^−4^). Among these, 7402 (82%) were downregulated and 1577 (18%) were upregulated in PT6 as compared to MPG (Fig. 4, Table 5 and Additional file 4: Table S3). To obtain information about functional differences between the MPG and PT6 transcriptomes, the differentially expressed unigenes were used to perform Gene Ontology (GO) enrichment analysis. Using R package topGO [35] with a cut-off of *P* < 0.01 and log2(Significant/Expected) >1, we identified a total of 78 enriched GO categories, of which 58 were enriched among upregulated unigenes and 22 were enriched among downregulated unigenes (Fig. 5). Interestingly, two GO categories (“cell wall”, “protein heterodimerisation”) were found to be enriched not only among upregulated DEGs, but also among downregulated DEGs, indicating that each of these two categories contains a substantial number of genes that are upregulated after pollen germination, along with many other genes that are downregulated in the course of the same process.

**Table 4:** Summary statistics for Illumina sequencing and unigene mapping

	MPG	PT6
Total number of clean reads	55,272,526	57,090,090
Average length of cleaned reads (nt)	98	98
Range of clean read lengths (nt)	40–100	40–100
Number of clean reads mapped to unigenes	54,123,863	55,961,167
Number of mapped unigenes	44,012	43,810

**Table 5:** Statistics of DEG analysis

	DEGseq
DEGs	8979
Upregulated DEGs	1577 (18%)
Downregulated DEGs	7402 (82%)

### Validation of differentially expressed unigenes

**Fig. 6:**
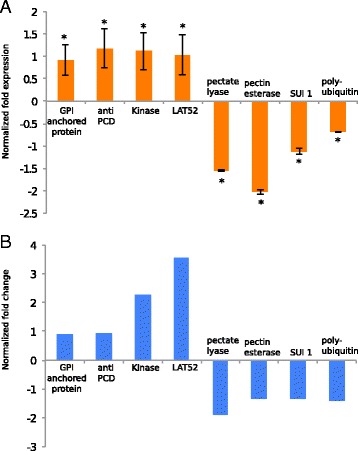
qRT-PCR analysis of differentially expressed unigenes. **a** qRT-PCR analysis of the expression of four unigenes each that are upregulated (left) or downregulated (right) in PT6 as compared to MPG. Normalized fold change: PT6 expression level /MPG expression level. *: *P* < 0.05, *n* = 6 replicates. Error bars, s.e.m. **b** DEGseq analysis of up- and downregulation of the same unigenes that were analyzed by qRT-PCR. Normalized fold change: PT6/MPG log2 values

To validate the detection of DEGs based on RNA-seq data, transcript levels of four unigenes each found to be substantially up- or downregulated in PT6 [normalized |log2(fold change)| ranging from 0.92 to 3.55] were analyzed by quantitative real-time PCR (qRT-PCR). Unigenes predicted to code for proteins with potential functions in processes essential for pollen tube tip growth were selected for this analysis. In all cases, the results of the DEGseq analysis were confirmed by qRT-PCR data (Fig. 6).

## Discussion

In this study, RNA-seq technology was employed to generate tobacco (*N. tabacum*) male gametophyte transcriptome sequence and expression level databases, which enable analyses of the genetic basis of pollen germination, pollen tube growth and to some extent fertilization. We assembled 454 sequencing reads representing the tobacco male gametophytic transcriptome based on both de novo and reference based approaches. As compared to the de novo assembly, the reference assemblies appeared to be substantially less accurate and more prone to assembly errors, which may be expected considering the large size and complexity of the tobacco genome (4.5 Gb and >70% repeat content) along with the fact that only draft sequences of this genome are currently available for reference assembly. All downstream analyses were therefore performed based on the de novo assembled transcriptome.

The de novo assembled transcriptome encompasses 76,364 unigenes, which likely represent both single transcripts per gene as well as multiple transcripts per gene (i.e. alternative splice variants). To determine the number of genes expressed in the tobacco male gametophyte we mapped our de novo assembly to the SGN tobacco unigene database with a total of 84,602 unique entries and found that our assembly represents 24,933 (29,5%) of these entries. Therefore, roughly 25,000 genes appear to be expressed in the tobacco male gametophyte, assuming that all unique entries in the SGN tobacco unigene database represent single genes. This number is substantially higher than previous estimates (~14,000–17,000) derived from analyses using the tobacco 44 k gene chip [21, 22], which is not surprising considering that this chip, which has been developed based on substantially older and less complete genome sequence databases, can only represent about 50% of the 81,000–94,000 genes identified in the recently published tobacco draft genome sequences [20]. Interestingly, despite these differences, both our RNA-seq and the previous gene chip analyses consistently indicate that approximately 30% of all tobacco genes are expressed in pollen and pollen tubes (gene chip: ~14,000–17,000 of ~44,000 probed genes; RNA-seq: 25,000 of 81,000–94,000 total genes). Similar percentages were also reported for Arabidopsis (29%, gene chip) [36] and maize pollen (37%, RNA-seq) [37].

Based on our de novo assembled male gametophytic transcriptome, we compared gene expression levels between two developmental stages: MPG and PT6. Illumina sequencing reads obtained from separate MPG and PT6 cDNA libraries both mapped to approximately 44,000 unigenes in the assembled transcriptome, indicating that a similar number of genes are expressed at the two different developmental stages. Even though equal numbers of genes appear to be transcribed at the MPG and PT6 stages, as many as 8979 unigenes were found to be significantly differentially expressed between these two stages, demonstrating that substantial transcriptional regulation occurs during pollen germination and early pollen tube growth. Interestingly, the majority of the identified DEGs are downregulated in PT6 relative to MPG. This pattern is expected if the activity of the transcription machinery is generally reduced after pollen germination. Thus, our results are consistent with previous findings indicating that mRNAs required for pollen tube growth accumulate during pollen maturation and that pollen tube growth early after germination displays low dependency on active transcription [12–17]. However, a substantial number of genes are in fact upregulated in PT6 relative to MPG and, interestingly, a particularly large number of overrepresented GO categories can be assigned to these upregulated genes. This indicates that although pollen germination is accompanied by a general, rather unspecific down regulation of gene expression, it also requires the specific activation of a subset of genes with important functions in elongating pollen tubes. In fact, many of the genes upregulated in PT6 belong to GO categories potentially playing important roles in pollen tube growth and/or fertilization, such as “pollen tube”, “pollen tube development”, “ruffle organization”, “regulation of unidimensional cell growth”, “regulation of actin filament depolymerization”, “pollen tube reception”, “synergid death and fertilization”. The identification of genes belonging to the last three GO categories (“pollen tube reception”, “synergid death” and “fertilization”) among those upregulated in PT6 appears particularly interesting, as the genetic control of processes involved in fertilization is currently only poorly understood [38]. Furthermore, the GO category RISC (“RNA-induced silencing”) complex was also overrepresented among the genes upregulated in PT6, which supports previous observations indicating that Arabidopsis miRNAs are regulating target genes with functions in male gametophyte development [39].

## Conclusions

To support an active area of ongoing research concerned with the characterization of cellular processes and regulatory mechanism underlying polar cell growth in plants, we have developed two high-quality tobacco databases, one of them representing a major proportion of the male gametophyte transcriptome and the other containing encompassing information about global changes in gene expression after pollen germination. A total of 76,364 unigenes representing the tobacco male gametophytic transcriptome were identified based on de novo assembly of 454 RNA-seq data. As indicated by the number of entries in the SGN tobacco database to which these unigenes could be matched, approximately 24,933 genes appear to be expressed in the tobacco male gametophyte, which correspond to roughly 30% of all genes in the tobacco genome. In addition, based on Illumina sequencing gene expression levels at the MPG and PT6 stages of tobacco male gametophyte development were globally quantified. Even though similar numbers of genes appear to be expressed at the MPG (44,012 matched reads) and PT6 (43,810 matched reads) stages, 8979 unigenes were found to be significantly differentially expressed between these two developmental stages. The majority of these unigenes (7402) were downregulated in PT6, indicating a general reduction of transcriptional activity after pollen germination. By contrast, a comparably small number of genes (1577) were upregulated in PT6. As expected, many of these genes are predicted to have specific functions in pollen tube growth and fertilization. In summary, this study advances the investigation of tobacco male gametophyte development by providing high-quality transcriptome sequence as well as expression level databases, and by enhancing current knowledge of the genetic control of this process resulting from quantitative global analyses of these databases.

## Methods

### Sample collection and RNA extraction

Tobacco (*N. tabacum* cv. Petit Havana SR1) pollen suspensions in liquid culture medium (200 anthers in 25 ml culture medium per sample) were prepared as described in Kost et al. [40]. For in vitro pollen germination and pollen tube growth, pollen suspensions were transferred to a 15 cm Petri dish and incubated at 25 °C in the dark. Pollen in suspension and pollen tubes grown for 3 and 6 h were collected by centrifugation and washed as previously described in Klahre et al. [41]. Pollen and pollen tube pellets were snap-frozen in liquid nitrogen for storage. Without grinding, total RNA was extracted using an RNeasy Plant Mini Kit (including DNase I treatment) according to the manufacturer’s instructions (Qiagen, Germany).

### Library preparation, 454 sequencing and de novo assembly

Equal amounts of tobacco male gametophytic mRNA isolated at the three developmental stages (MPG, PT3 and PT6) were pooled and used for the construction of a single normalized random-primed cDNA library, which was sequenced based on the Roche/454 GS FLX+ technology at Eurofins MWG (Germany). Raw 454 sequencing reads were pre-cleaned (removal of adapters and short sequences with low quality scores) by Eurofins MWG and subsequently de novo assembled using MIRA v 3.4 [24] with default parameters for four cycles, during which contigs and singletons from the previous cycles were merged and used as input sequences in the subsequent cycles. Finally, the contigs and singletons obtained after the fourth and final round were merged and clustered by CAP3 [25]. In the final assembly, only sequences larger than 80 nt were included (unigenes).

### Reference assembly

Three draft *Nicotiana tabacum* genomes were downloaded from the DDBJ/EMBL/GenBank nucleotide core database using the accession codes AYMY00000000 (TN90), AWOJ00000000 (K326) and AWOK01000000 (Basma Xanthi). Our 454 sequencing reads were first aligned to the reference genomes using BLAT, before the resulting alignments were used for transcriptome assembly by Cufflinks [28] with default parameters.

### Analyses of the number of expressed genes and completeness of the de novo assembled transcriptome

In order to estimate the number of genes expressed in tobacco pollen and pollen tubes, assembled unigenes were queried against the Sol Genomics Network (SGN) tobacco unigene database (https://solgenomics.net) [42] with a threshold E-value of 10^−5^. To learn more about how completely the assembled unigenes cover the tobacco male gametophyte transcriptome, the unigenes were first translated to peptide sequences with EMBOSS Transeq [43] using all six reading frames. Then eukaryotic ultra-conserved orthologs (UCOs) among the translated unigenes were determined by BLAT using a threshold E-value of 10^−10^ against a list of 357 UCO protein sequences from Arabidopsis (sequences available at: http://compgenomics.ucdavis.edu/compositae_reference.php). Finally, the translated unigenes were compared against the PlantTribes database [30, 32]. In this analysis, 983 shared single copy tribes from *Arabidopsis thaliana, Populus trichocarpa, Vitis vinifera* and *Oryza sativa* were compared with the translated unigenes using tblastx and an E-value cutoff of 10^−10^. In all analyses, only the best matches (highest bit-score) were kept for each query sequence.

### Identification of full-length cDNA

Putative full-length cDNAs were identified among the identified unigenes using the web based tool TargetIdentifier [33]. In TargetIdentifier, a cDNA sequence was recognized as a full-length cDNA if the start codon (ATG) and poly (A) tail were identified.

### Comparison of gene expression in tobacco pollen and pollen tubes

Two separate 3′-fragment cDNA libraries were constructed from tobacco male gametophytic mRNA isolated either at the MPG or PT6 stage of development. Each of these two libraries was sequenced using an Illumina HiSeq 2000 instrument at Eurofins MWG (Germany). Gene expression was quantified based on the sequence coverage of the Illumina sequencing reads that were mapped by BLAT to the de novo assembled unigenes with a cut-off E-value of 10^−5^. Only the top match with highest bit-score for each sequencing read was kept. To identify unigenes differentially expressed between MPG and PT6 (DEGs), we summarized the Illumina RNA-seq read coverage of each unigene and DEGs were identified using the R package DEGseq [34] with a threshold of q-value ≤0.0001. The Gene Ontology (GO) enrichment analyses of DEGs were performed using the topGO R package with fisher’s exact test and a cut-off *P* < 0.01 and log2(Significant/Expected) >1.

### qRT-PCR

To confirm the detection of differential gene expression based on the RNA-seq technology, quantitative real-time PCR (qRT-PCR) was performed on eight DEGs. Four of these DEGs were upregulated in PT6 compared to MPG (LAT52, GPI-anchored protein LORELEI, anti PCD, and protein kinase-like protein), whereas the other four were downregulated (SUI 1, polyubiquitin, pectate lyase and pectin esterase). Primers were designed using the Perlprimer software [44] to obtain melting temperatures (Tm) ranging from 59 to 61 °C and fragment sizes from 100 bp to 250 bp. MPG and PT6 mRNA was isolated as described above. Per sample, 1 μg mRNA was reverse transcribed using an iScript™ cDNA Synthesis Kit (Bio-Rad) according to the manufacturer’s instructions, and subsequently diluted 250× in nuclease free water. Real time quantitative PCR (qRT-PCR) was performed in a 96 well thermocycler (C1000 Touch™ Thermal Cycler, CFX96™ Real-Time System, Bio-Rad) using iQ™ SYBR® Green Supermix (Bio-Rad). The thermocycler was programmed to run for 3 min at 95 °C, followed by 45 cycles of 10 s at 95 °C and 30 s at 60 °C. RNA isolated from six identically treated MPG and PT6 samples were analyzed to account for biological variation and each of the six biological replicates was run twice on the thermocycler to correct for technical variation. Genomic DNA extracted from tobacco leaves using a DNeasy Plant Mini Kit (Qiagen) was serially diluted to concentrations ranging from 50 to 64,800 estimated genome copies per well and used to test amplification efficiency. Specific amplification of single fragments was confirmed by recording a melting curve upon heating each PCR product from 60 °C to 95 °C. Relative expression levels of each gene were calculated using qbasePLUS software (Biogazelle), based on two reference genes for data normalization (NtL25 and NtGAP1).

## Additional files

Additional file 1: Sequences of all unigenes in FASTA format. (ZIP 16486 kb)

Additional file 2: Table S1. Unigene Annotation based on the SGN tobacco unigene database. (XLSX 6422 kb)

Additional file 3: Table S2. Full-length cDNAs identified using TargetIdentifier. (XLSX 4144 kb)

Additional file 4: Table S3. Differentially expressed unigenes identified by DEGseq. (XLSX 1211 kb)

## Abbreviations

DEGs: Differentially expressed genes
FDR: False discovery rate
GO: Gene ontology
MPG: Mature pollen grain
Nt: Nucleotides
PT3: Pollen tube grown for three hours
PT6: Pollen tube grown for six hours
qRT-PCR: Quantitative real-time PCR
RNA-seq: RNA sequencing
